# Building Capacity in Perioperative Quality Improvement in Low‐ and Middle‐ Income Countries: Experiences From Mombasa, Lusaka and Hawassa

**DOI:** 10.1002/wjs.70126

**Published:** 2025-10-09

**Authors:** Anoosha Moturu, Sehar Salim Virani, Taylor Jaraczewski, Belay Melesse, Tien Vo, Clifford Y. Ko, Francis Pikiti, Hazel Sonkwe, Bright Moyo, Kelly McQueen, Girma Tefera, Xane Peters, Daniel Ojuka, Haytham Kaafarani, Robert Parker, Chris Dodgion, Deborah Rusy, Syed Nabeel Zafar

**Affiliations:** ^1^ American College of Surgeons Division of Research and Optimal Patient Care Chicago Illinois USA; ^2^ Division of Surgical Oncology Department of Surgery UW Carbone Cancer Center University of Wisconsin School of Medicine and Public Health Madison Wisconsin USA; ^3^ Department of Surgery Medical College of Wisconsin Milwaukee Wisconsin USA; ^4^ Hawassa University Comprehensive Specialized Hospital Hawassa Ethiopia; ^5^ University Teaching Hospital Lusaka Zambia; ^6^ School of Medicine University of Zambia Lusaka Zambia; ^7^ Department of Surgery University of Wisconsin Madison Wisconsin USA; ^8^ American College of Surgeons Health Outreach Program for Equity in Global Surgery Chicago Illinois USA; ^9^ University of Nairobi Nairobi Kenya; ^10^ Division of Trauma Emergency Surgery & Surgical Critical Care Department of Surgery Massachusetts General Hospital Boston Massachusetts USA; ^11^ Tenwek Hospital Bomet Kenya

**Keywords:** capacity building, low‐ and middle‐income countries, perioperative education, quality improvement

## Abstract

**Background:**

Perioperative morbidity and mortality remain disproportionately high in low‐ and middle‐income countries (LMICs). Quality improvement (QI) has shown effectiveness in improving health outcomes and patient safety across healthcare systems globally and is often a mandatory part of training in high income countries. However, QI education in LMICs remains decentralized and limited in scope. Aiming to address this gap, we designed and delivered perioperative QI education in three LMIC settings. This study aims to summarize methodology and experiences across the three settings and outline the factors leading to success and challenges experienced to inform future QI education efforts in LMICs.

**Methods:**

We designed and implemented a perioperative QI curriculum that included online didactic content, course assessments, and a two to three day in‐person course featuring both didactic and QI project development training. Descriptive statistics were reported for course features and participant pre‐ and post‐course survey results. Qualitative course feedback from participants and administrators is summarized in a narrative format.

**Results:**

Five courses were conducted, training a total of 90 participants. Of those who completed pre‐course surveys, most participants were nurses (35.5%) or residents (43.6%), and the majority (71.1%) had no prior experience with QI. Self‐assessed comfort significantly improved across several domains, including understanding QI concepts, developing aim statements, and designing projects. Qualitatively, participants enjoyed the course's interactive format and recommended future offerings be longer, more frequent, and include expanded content on data analysis and project implementation. Course administrators recommend involving local partners throughout course development, forming structured plans to sustain QI initiatives started as part of the course, and ensuring any pre‐course materials use context‐specific examples.

**Conclusion:**

Implementation of a perioperative QI curriculum across three LMIC settings was feasible and associated with significant improvements in participant‐reported comfort with core QI skills and knowledge. Success was driven by the inclusion of local faculty, interactive project development, and adaptation of course content to local context. Future efforts should focus on building local QI mentorship capacity, integrating QI education into residency training, and developing contextually appropriate resources to support scalable, sustainable QI training in LMICs.

## Graphical abstract

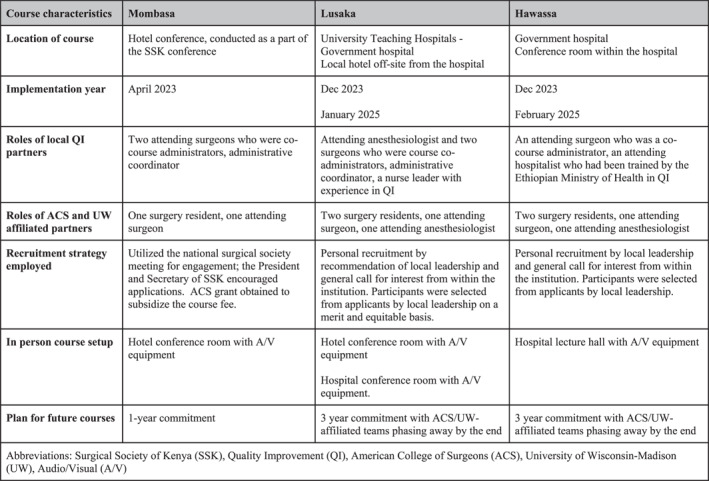

## 1 Background

The Quality Improvement (QI) movement, which gained significant momentum in the 1990s [1], has shown effectively improved health outcomes and patient safety across healthcare systems globally [2]. Core QI principles, such as the Donabedian model, emphasizing structure, process, and outcome, offer valuable frameworks for implementation in diverse settings, including resource‐constrained environments [3, 4, 5]. Despite broadly applicable QI frameworks, LMICs often face barriers such as resource constraints and inadequate infrastructure. Additionally, due to a shortage of healthcare professionals trained in QI, many programs depend heavily on external collaborators, resulting in short‐term, unsustainable initiatives [6, 7]. Increasingly, the importance of locally developed and led QI in LMICs is being recognized. Programs like Lifebox, which provides empirical perioperative QI education [8, 9, 10], and initiatives by the Ethiopian Ministry of Health promoting national QI education, aim to address this gap [11]. These efforts demonstrate the potential of context‐specific QI education to generate impactful local initiatives.

A specific area for QI in LMICs is perioperative morbidity and mortality, which remains disproportionately high [12]. In United States, programs like the National Surgical Quality Improvement Program (NSQIP), and the Anesthesia Quality Institute (AQI)/National Anesthesia Clinical Outcomes Registry (NACOR) have enhanced perioperative quality [13]. Perioperative QI education is encouraged through the Accreditation Council for Graduate Medical Education requirements for resident education, institutional QI task forces, and the American College of Surgeons (ACS) QI curricula [14]. Similar efforts worldwide, including the Australian and New Zealand Audit of Surgical Mortality (ANZASM), the NHS National Clinical Audits, and the European Surgical Outcomes Study (EuSOS), have likewise contributed to improvements in perioperative outcomes [15, 16]. In contrast, QI education in LMICs may be decentralized or centrally managed at the national level without effective dissemination. To address this, we designed and delivered perioperative focused QI education in LMICs. Here, we share our experiences from 3 different countries.

This paper describes the implementation of this course in three LMIC settings: Mombasa (Kenya), Lusaka (Zambia), and Hawassa (Ethiopia), highlighting success factors and challenges experienced to inform future QI education efforts in LMICs.

## 2 Methods

This study is a review of the implementation of perioperative QI curriculum in three LMIC settings which occurred June 2022–December 2023. This initiative represented a collaborative effort among faculty and staff from the University of Wisconsin‐Madison (UW), Tenwek Hospital (TH), University of Nairobi (UN), the University Teaching Hospital, University of Zambia (UTH‐UNZA) in Lusaka, Zambia, Hawassa University Comprehensive Specialized Hospital (HU), Hawassa, Ethiopia. In collaboration with the ACS Health Outreach Program for Equity in Global Surgery (H.O.P.E), the ACS International Relations Committee, the Surgical Society of Kenya, the Global Academic Anesthesia Consortium (GAAC), and the Society of Anesthetists of Zambia, with funding from the University of Wisconsin Reilly‐Baldwin Wisconsin Idea award, and the Pon Fund from the ACS International Relations Committee.

### 2.1 Curriculum Development

The curriculum combined online didactics, course assessments, and a two‐to‐three‐day in‐person course featuring both didactic sessions and project development. It covered QI theory, methodology, and real‐world examples from both LMIC and HIC contexts. Course content was co‐developed by UW, ACS, and local faculty (at TH, UN, UTH‐UNZA, and HU) and tailored for each site. The in‐person course ended with project development tasks in multidisciplinary small teams of five to seven participants, where teams identified healthcare challenges and developed actionable QI project plans under instructor mentorship. Each team was guided to develop key deliverables, including a problem statement, aim statement, a root cause analysis diagram (e.g., fishbone diagram, key driver diagram, five why exercise), and a rough project timeline.

The curriculum was based on the ACS QI Basics Course, originally designed for US‐based healthcare professionals, offering a comprehensive foundation in perioperative quality and safety. It includes six modules: Introduction to QI, The QI Process, Data Measurement and Analysis, Change Management, Patient Safety, and Leadership and Teamwork for QI [17]. Recognizing the importance of local relevance, we adapted the curriculum to include context‐specific content and local mentorship. For example, though the core concept of Plan, Do, Study, Act cycles were covered at each site, the presentation slides may have varied at the preference of a local course instructor. Additional sessions were included when relevant, such as one by a UTH trainee sharing successful QI projects from their hospital. Thus, all ACS QI content was delivered, supplemented by local, context‐specific enhancements.

### 2.2 Course Assessments

Pre‐ and post‐course surveys assessed participants' baseline knowledge and comfort with QI concepts. These paper or electronic surveys used Likert‐scale questions [1, 2, 3, 4, 5] and included demographic questions. Post‐course assessments focused on changes in self‐assessed skills and session effectiveness. Course structure and assessments were adapted per site, in collaboration with local faculty as described below.

### 2.3 Mombasa

Participants were surgeons attending the annual Surgical Society of Kenya (SSK) meeting, recruited via open applications. SSK selected a diverse group based on sex, region, specialty, experience, and leadership potential. Participants were required to complete the ACS online modules and pass module assessments before attending the 2‐day in‐person course, which included all core didactics and project development sessions. Teams developed draft QI projects relevant to their settings. Local facilitators provided country‐specific examples and framed discussions for contextual relevance.

### 2.4 Lusaka

In year one, participants were nominated by local leadership; in year two, they were selected from open applications reviewed on merit and equity. The cohort included trainees, staff, and faculty from surgery, anesthesia, emergency medicine, and nursing. The ACS QI online modules were provided to participants for voluntary completion. The in‐person sessions covered all ACS topics and were supplemented by talks from UTH faculty and Zambia's Ministry of Health, emphasizing the feasibility of QI in local contexts. Presenters included physicians, nurses, and trainees who shared successful QI projects. Project development sessions were held on the final day.

### 2.5 Hawassa

Recruitment followed Lusaka's model, with participants from surgery, anesthesia, emergency medicine, perioperative nursing, operating room pharmacy, and lab staff. The ACS QI Basics Course online modules were offered for voluntary completion. Didactics were condensed into 2 days using the Ethiopian Ministry of Health's 5‐day curriculum and included all ACS topics. The final day focused on project development, applying QI principles to local healthcare issues.

### 2.6 Study Analysis

#### 2.6.1 Course Features and Participant Survey Results

Descriptive statistics, including frequencies, percentages, and means, were reported for course features and participant survey results. Course features included implementation year, QI background and roles of local and ACS‐affiliated course administrators, recruitment strategy, in‐person course setup, plan for future courses, and lecture topics. Survey results included participant demographics, pre‐ and post‐course assessments of self‐assessed QI comfort, and session effectiveness. The participant survey results only reflect participants from the Lusaka and Hawassa courses. Differences in pre‐ and post‐course median scores were analyzed using Wilcoxon rank sum tests.

### 2.7 Qualitative Feedback From Participants and Course Administrators

Post‐course assessments at all sites included open‐ended feedback from participants, summarized narratively. After the courses were administered, course administrators (directors, and key faculty) were invited to share their qualitative feedback on factors leading to success and challenges experienced throughout the process. This feedback was collected through informal conversations, summarized, and reviewed by all administrators for accuracy and completeness.

### 2.8 Ethics Approval

This manuscript describes our experience with delivering educational content. Based on guidance from the University of Wisconsin Institutional Review Board, the work is not considered human subjects research and therefore did not require IRB approval.

## 3 Results

### 3.1 Course Features

The courses were conducted in three LMIC settings: Mombasa, Kenya (April 2023), Lusaka, Zambia (December 2023 and January 2025), and Hawassa, Ethiopia (December 2023, and February 2025). Each course was delivered by a combination of local and ACS‐affiliated instructors with varying levels of QI expertise (Table 1). Recruitment strategies varied across sites and included leveraging national surgical society meetings, departmental invitations by hospital administrators, and personal outreach. Courses were conducted in hotel or hospital conference rooms equipped with audiovisual tools to facilitate presentations and group discussions.

**TABLE 1:** Course characteristics.

Course characteristics	Mombasa	Lusaka	Hawassa
Location of course	Hotel conference, conducted as a part of the SSK conference	University teaching hospitals—government hospital Local hotel off‐site from the hospital	Government hospital Conference room within the hospital
Implementation year	April 2023	Dec 2023 January 2025	Dec 2023 February 2025
Roles of local QI partners	Two attending surgeons who were co‐course administrators, administrative coordinator	Attending anesthesiologist and two surgeons who were course co‐administrators, administrative coordinator, a nurse leader with experience in QI	An attending surgeon who was a co‐course administrator, an attending hospitalist who had been trained by the Ethiopian ministry of health in QI
Roles of ACS and UW affiliated partners	One surgery resident, one attending surgeon	Two surgery residents, one attending surgeon, one attending anesthesiologist	Two surgery residents, one attending surgeon, one attending anesthesiologist
Recruitment strategy employed	Utilized the national surgical society meeting for engagement; the President and Secretary of SSK encouraged applications. ACS grant obtained to subsidize the course fee.	Personal recruitment by recommendation of local leadership and general call for interest from within the institution. Participants were selected from applicants by local leadership on a merit and equitable basis.	Personal recruitment by local leadership and general call for interest from within the institution. Participants were selected from applicants by local leadership.
In person course setup	Hotel conference room with A/V equipment	Hotel conference room with A/V equipment Hospital conference room with A/V equipment.	Hospital lecture hall with A/V equipment
Plan for future courses	1‐year commitment	3 years commitment with ACS/UW‐affiliated teams phasing away by the end	3 years commitment with ACS/UW‐affiliated teams phasing away by the end

Abbreviations: A/V: Audio/Visual, ACS: American College of Surgeons, QI: Quality Improvement, SSK: Surgical Society of Kenya, UW: University of Wisconsin–Madison.

The structure of the courses reflected site‐specific plans for sustainability. In Mombasa, the course was planned as a one‐time event during the Surgical Society of Kenya meeting with a focus on training the trainers and leaders. In contrast, course administrators in Lusaka and Hawassa adopted a phased approach, with three annual course cycles planned to progressively transition leadership to local teams. This approach incorporated mentorship and exchange programs to ensure sustainable QI education efforts and reduce reliance on external facilitators. A total of 18 didactic topics were covered, including broadly relevant content such as *Introduction to QI* and *Data Measurement and Analysis*, as well as locally relevant topics like *Connecting QI to National Key Performance Indicators* (Table 2).

**TABLE 2:** Lecture topics.

Lecture topics included	Mombasa	Lusaka	Hawassa
The Fundamentals of QI			
Introduction to QI: Donabedian model and domains of quality	X	X	X
Patient safety	X	X	X
Improving patient flow for obstetrics patients in Zambia at women and newborn hospital		X	
Incorporating QI in residency		X	X
The QI process: Science of improvement	X	X	X
Introduction to clinical audits		X	X
Overview of the ACS NSQIP program		X	X
Leadership and teamwork for QI	X	X	X
Perioperative registry building and using data		X	X
Overview of national KPIs—in surgery and anesthesia		X	X
Examples: Example of QI in surgery US based—SSI reduction, post whipple infections and NSQIP data, QI in anesthesia		X	
Getting and maintaining buy in	X		
Starting your QI project			
Applying the concepts to your practice	X		
Models and problem identification		X	X
Concepts and methods and problem and aim statement		X	X
Data measurement and analysis	X	X	X
Change management, measurement, and testing of change	X	X	X
Sustainability, scaling up and spreading			X
Completing a QI project	X		
Publishing a QI project		X	X

Abbreviations: ACS: American College of Surgeons, KPI: Key Performance Indicators, NSQIP: National Surgical Quality Improvement Program, PDSA: plan do study act, QI: Quality Improvement, SSI: surgical site infection, US: United States.

### 3.2 Participant Survey Results

In Mombasa, 30 participants completed the course, including 24 surgeons, 4 residents, and 2 medical students. Of the 29 participants who responded to the post‐course survey, all indicated they would recommend the course to others. Participants reported a wide range of time spent on the pre‐course online material, with a median of 20 h (range: 8–120 h). The in‐person course was rated as *excellent* by 66%, *very good* by 28%, and *good* by 7% of respondents. The online course was rated *excellent* by 61%, *very good* by 25%, *good* by 7%, and *fair* by 7%. All respondents reported that the course was relevant to their context, that facilitators explained content clearly, and that they felt comfortable voicing their opinions. Additionally, 90% of participants expressed interest in serving as future trainers, with the remaining 10% responding “maybe.”

In Lusaka and Hawassa, a total of 90 participants (41 from Lusaka, 49 from Hawassa) completed pre‐workshop surveys and 68 participants completed post‐workshop surveys (41 from Lusaka, 27 from Hawassa). The majority of participants were nurses (35.6%) and residents (43.6%) from the Surgery (48.9%) or Anesthesia (25.6%) departments (Table 3). The majority had not participated in a QI project before (71.1%). Median scores of self‐assessed QI comfort increased significantly from pre‐to post‐course for understanding of QI concepts (3 [IQR 2–3] to 5 [IQR 4–5], *p* < 0.001), developing a QI aim statement (3 [3–5] to 4 [4–5], *p* < 0.001), designing a QI project (3 [2–5] to 4 [4–5], *p* < 0.001), and interpreting data for a QI project (3 [IQR 2–5] to 4 [IQR 4–5], *p* < 0.001) (Table 4).

**TABLE 3:** Demographic characteristics of participants of the quality improvement (QI) courses.

Characteristics	*N* (%)
Current position in hospital	
Nurse	32 (35.6)
Resident/Trainee	39 (43.6)
Faculty	15 (16.7)
Other	4 (4.4)
Department	
Surgery	44 (48.9)
Anesthesia	23 (25.6)
Critical care and emergency	8 (8.9)
Others	5 (5.6)
Missing	10 (11.1)
Age	
26–30	28 (31.1)
31–40	56 (62.2)
41–50	3 (3.3)
51+	3 (3.3)
Sex	
Female	34 (37.8)
Male	56 (62.2)
Have you participated in a QI project before?	
No	64 (71.1)
Yes	26 (28.9)

*Note:* The results here are from course participants from the Hawassa and Lusaka courses, as they had the same course assessments.

**TABLE 4:** Pre‐ and post‐ course assessments of self‐assessed QI comfort.

Question	Pre‐workshop score Median (IQR)	Post‐workshop score Median (IQR)	*p* value
Do you understand basic concepts of QI?	3 (2–4)	5 (4–5)	**< 0.001**
How important do you think QI is to the work you do?	5 (5–5)	5 (5–5)	0.8438
Are you interested in learning more about QI?	5 (5–5)	5 (5–5)	1.0000
Do you feel the need for mentorship in QI?	5 (4–5)	5 (4–5)	0.5621
Rate your comfort level with developing a QI aim statement	3 (3–5)	4 (4–5)	**< 0.001**
Rate your comfort level with designing a QI project	3 (2–5)	4 (4–5)	**< 0.001**
Rate your comfort level with interpreting data for QI project	3 (2–5)	4 (4–5)	**< 0.001**
Over the next 12 months, how likely are you to design and/or participate in a quality improvement project?	4 (4–5)	5 (4–5)	**0.0082**
Over the next 12 months, how likely are you to lead in a quality improvement project?	4 (3–5)	4 (4–5)	0.2244

*Note:* The results here are from course participants from the Hawassa and Lusaka courses, as they had the same course assessments. There were a total of 52 pre‐workshop responses (26 at HUSCH and 26 at UTH) and 30 post‐workshop responses (26 at HUSCH and 4 at UTH). The above table shows mean, mean difference and *p* value for the 30 matched pre‐ and post‐workshop responses. Scores based on a 5‐point Likert scale (1 = not at all, 5 = very much). Wilcoxon signed‐rank test used for paired comparisons.

### 3.3 Examples of QI Projects Developed

During the workshops, participants formed small teams and worked through locally relevant problem statements to design potential QI projects. The projects addressed diverse issues across surgical and perioperative care, ranging from improving infection prevention practices to optimizing preoperative evaluation and reducing unnecessary hospital stay. Examples of protocols developed during the workshops include improving preoperative patient evaluation, enhancing infection prevention during delivery, implementing sedation guidelines in intensive care, and strengthening staff engagement to improve patient satisfaction.

### 3.4 Qualitative Feedback From Participants

The post‐course feedback indicated a positive response from participants. The majority of attendees found the course engaging and reported an improved understanding of QI concepts due to the facilitators' ability to simplify content and address queries with patience. They also stressed the importance of the interactive project development portion of the course to elucidate the didactic contents through applied learning and highlighted the benefit of initiating QI project design during the workshop itself in multidisciplinary teams.

In addition, several areas for improvement were identified through participant feedback. First, participants suggested more checkpoints to stimulate progress for QI projects that were started as part of the course. They suggested holding QI courses 2–3 times per year, establishing a monthly virtual community of practice to sustain mentorship, and providing structured support for ongoing projects. Second, the 2‐day course was perceived as too short, and participants suggested extending it to three days or 1 week to allow deeper discussion of data management, change management, and stakeholder engagement. Third, requests were made for more detailed sessions on data analysis, presentation techniques, and how to obtain necessary permissions to conduct QI projects within hospital settings. They also requested more examples from local hospitals, practical case studies, and strategies for effectively pitching QI projects to leadership. Finally, participants emphasized the need for continuous mentorship and improved resource allocation to facilitate QI project implementation beyond the course setting. Additional qualitative feedback on individual sessions revealed that participants generally found sessions to be useful, with an appropriate amount of information, and effective speakers (Table S1).

### 3.5 Qualitative Feedback From Course Administrators

Course administrators learned from both factors which contributed to the success of the courses and the challenges experienced. These factors led to several recommendations for future QI courses administered in LMIC settings (Table 5). These recommendations include involving local faculty early in course development, recruiting multidisciplinary participants, and identifying highly engaged individuals to serve as future mentors or facilitators. Simplified and context specific pre‐course materials for participants should be required, when possible, as they can help establish a shared foundation of material for participants. During the in‐person course, structured small‐group project development sessions with clear deliverables should be incorporated. To enhance project continuity, small teams should be formed in advance (ideally from the same institution) and supported with structured post‐course mentorship and accountability mechanisms. To further support project continuity, efforts should be made toward strengthening digital data tracking systems and including IT professionals in QI teams. During the course, additional sessions on how to how to engage hospital leadership and navigate institutional processes should be incorporated. To sustain course delivery over time, a phased, multi‐year implementation model (such as those used in Lusaka and Hawassa) can gradually transition leadership to local teams. Finally, long‐term engagement may be supported through institutional and national policy advocacy and incentives such as continuing professional development credit, authorship opportunities, and protected time.

**TABLE 5:** Recommendations for future QI courses in LMIC settings.

Success or Challenge	Description	Recommendation
Success: Local leadership	Local instructors enhanced relevance by adapting examples and facilitating discussion grounded in local realities.	Involve local faculty from the outset of course development to ensure content is context‐specific and fosters participant trust and engagement.
Success: Interactive project development	The group‐based QI project development sessions fostered multidisciplinary collaboration and practical application of QI principles.	Incorporate small‐group project design sessions with clear deliverables and mentorship into courses to reinforce hands‐on learning.
Success: Phased implementation	The phased approach in lusaka and Hawassa provided a structured transition of leadership to local teams over three years to promote the long‐term sustainability of QI efforts.	Structure future courses with a multi‐year mentorship model, including a gradual transition of course leadership to local instructors.
Success: Capacity building of local surgical workforce	Training a diverse group of clinicians (including nurses, residents, and consultants) helped establish institutional QI champions.	Actively recruit a multidisciplinary mix of participants to build peer‐to‐peer QI mentorship within institutions.
Success: Identification of QI opportunities	Courses surfaced practice gaps and created opportunities for QI.	Encourage participants and faculty to identify local challenges as potential QI opportunities.
Success: Strong Senior surgeon engagement	Senior clinicians were actively involved and many expressed interest in facilitating future trainings.	Identify and support highly engaged participants as future course facilitators or local QI mentors to promote sustainability.
Success: Required pre‐course work	Participants who completed pre‐course modules had stronger baseline knowledge and participated more fully in discussion.	When possible, require completion of pre‐course didactic content when feasible to ensure shared foundational understanding and more productive in‐person sessions.
Challenge: Limited administrative support	Course coordination and participant communication required significant effort without dedicated administrative staff.	Assign a local course administrator to manage logistics, communication, and scheduling before and during the course.
Challenge: QI project sustainability	Projects were often not sustained, possibly due to institutional barriers, lack of mentorship, or dispersed teams.	Form teams from the same institution where possible, solicit project ideas prior to the course, and establish post‐course mentorship or peer learning communities. Keep the focus on institutional and local society capacity building to encourage project sustainability.
Challenge: Gaps in course content	Participants requested more guidance on aligning QI efforts with hospital leadership and navigating institutional processes.	Add sessions on engaging leadership, obtaining project approvals, aligning QI work with institutional goals, and others per request from participants.
Challenge: Online course completion	Despite interest, many participants did not complete the online modules, potentially due to time constraints and perceived lack of relevance.	Consider making some pre‐course content mandatory prior to in‐person portion. Simplify and contextualize online content for LMIC settings and provide estimates of time commitment along with non‐internet‐based alternatives if needed.
Challenge: Lack of electronic data platform	Collecting accurate and complete data to use to identify improvement efforts and monitor progress is challenging with paper records.	Wherever possible, work toward developing electronic data platforms to track patient data. Expand quality teams to include members from the computer science and engineering teams
Challenge: Lack of structural support in the form of equipment and personnel resources	Equipment shortages, staffing constraints, and bureaucratic delays hindered QI implementation.	Advocate for inclusion of QI training and support in national health and education policies to secure consistent institutional backing.
Challenge: Faculty and trainee engagement	Competing responsibilities limited engagement unless explicitly incentivized.	Offer continuing professional development (CPD) credit, authorship on resulting publications, and protected time to incentivize participation by local faculty and trainees.

## 4 Discussion

We implemented two‐to‐three‐day perioperative QI courses tailored to the LMIC context in Mombasa, Lusaka, and Hawassa. These were well received, feasible, and effective at training perioperative QI concepts to a wide range of participants.

### 4.1 Context and Barriers to QI in LMICs

Previous studies support the value of QI training to improve care in LMICs [18]. For example, the HEALTHQUAL program in Namibia partnered with the Ministry of Health to train clinical teams in local data‐driven QI methods and build national coaching capacity [19]. In another example, local teams provided structured on‐the‐job training in a Kenyan neonatal unit to improve documentation and antibiotic prescribing appropriateness [20]. Despite several examples of successful QI, challenges remain. These barriers include limited physical, technological, and human resources; inadequate systems for collecting and analyzing data; inconsistent QI funding; turnover or instability in institutional or national leadership that prioritizes QI work [21]; delays in ethics committee approval due to misclassification of QI as research; limited generalizability of QI interventions across settings; low rates of publication and dissemination of QI work; inadequate training and lack of QI in medical curricula; and lack of institutional incentives or professional recognition for QI work. While our collaborators and course participants also noted similar barriers, they also noted key drivers of success.

### 4.2 Role of Local Expertise

A key driver of success was the incorporation of local expertise in both content delivery and course facilitation. Local course directors and faculty were identified through hospital partnerships, professional networks, and word‐of‐mouth recommendations from national health ministries and QI champions. In addition to contextualizing course content with relevant examples, local speakers helped build credibility and inspiration among participants. Their contributions also enabled the integration of national key performance indicators and institutional priorities into the course structure.

### 4.3 Adaptation of Online Resources

This experience also highlighted a resource gap: the need for a version of the online course tailored specifically to LMIC settings. Recognizing this limitation, we initiated collaborative efforts with ACS and local partners to develop a modified, LMIC‐adapted version of the online curriculum including resource‐specific examples. The LMIC‐adapted version is currently undergoing pilot testing and will be integrated into future course offerings. Although the online course was offered at all sites, uptake was low in Lusaka and Hawassa where it was optional. In Mombasa, where completion of the course was required, participants engaged more deeply. Internet access and time constraints remain barriers to web‐based components, and future courses must plan for these challenges.

### 4.4 Long‐Term Sustainability of QI Initiatives

In Hawassa and Lusaka, we noticed increased acceptance and enthusiasm from several departments and hospital leadership for the QI course. This cultural shift happened in a short amount of time and led to participants and hospital leadership who are eager to facilitate QI education. While the content of this QI education was successfully delivered by the courses, one of the most significant challenges identified was the sustainability of the QI projects. Although participants actively developed and refined project ideas during the interactive project development sessions, there was a lack of robust strategies to support these projects beyond the course. Specifically, the following strategies may have facilitated greater sustainability beyond the course: ongoing mentorship, follow‐up meetings, and creating a platform for participants to collaborate and share progress after the course. Without these support structures, many project ideas risk remaining conceptual rather than translating into impactful initiatives.

To address these challenges, sustainability has been at the forefront of our program design moving forward. We are deliberately reducing external involvement with each successive workshop, with the goal of reaching 100% local faculty‐led delivery by the fourth year. Local QI champions are being developed through mentorship and bilateral exchange, including observerships at our partner hospitals in the United States to directly observe NSQIP processes and institutional QI culture. In parallel, we are establishing local data repositories and supporting prospective data collection using REDCap, carried out by staff hired and financially sustained by host institutions. Online teaching materials are also being created to supplement in‐person training and broaden access. Importantly, we aim to track impact through registry‐based assessments of patient‐level outcomes, ensuring objective evaluation of progress. A key priority is the integration of QI training into formal residency curricula, particularly in surgery and anesthesia, so that QI becomes embedded in professional training rather than dependent on external courses. This structured teaching has begun within the anesthesia training program at UTH in Lusaka. As local faculty and institutions gain expertise, the pool of mentors and champions will expand, providing the support structures needed for projects to transition from ideas to impactful, sustainable initiatives.

### 4.5 Importance of Data Infrastructure in LMICs

Ensuring quality data collection in LMICs is crucial for improving evidence‐based decision‐making in population health. Challenges for accurate data collection include limited resources and hospital infrastructure, including lack of electronic methods for capture, database platforms, and trained personnel, as well as data fragmentation between different organizations. To improve data quality, LMICs may invest in capacity building, digital data collection infrastructure, and personnel trained in and supporting QI.

### 4.6 Study Limitations

The results of this study should be interpreted in the context of its limitations. First, the reliance on qualitative data from participant surveys and feedback from course administrators may introduce bias. Second, the results may not apply universally to all LMICs due to differences in healthcare systems, resources, and institutional capacities across various regions. Third, the absence of long‐term follow‐up data restricts our capacity to assess the sustainability and impact of the QI projects initiated during the courses. While improvements in participant knowledge and self‐assessed comfort with QI were observed, this study did not evaluate whether training translated into measurable improvements in patient‐level outcomes such as morbidity, mortality, or efficiency of care. We are however, working with certain institutions on a perioperative outcomes registry to assess and evaluate surgical outcomes over time [22]. Once the data is more mature, we will be able to assess differences in outcomes over the years, however it will not be possible to make a causal assumption between this teaching and improvement in outcomes as we believe that observed improvements are likely to have many contributing factors. Although we delivered the same core content across all sites, there was variability in additional course topics, content delivery, and the involvement of administrators, which may have affected the consistency of participant experiences and outcomes. Finally, structural challenges, such as limited access to internet or administrative support, were not systematically addressed and could have influenced the effectiveness of the interventions. Despite these limitations, we hope that by sharing our experiences, we contribute to the growing body of knowledge on QI education in LMICs and provide insights to inform future QI training programs tailored to LMICs.

## 5 Conclusion

The successes and challenges we learned from while administering the QI courses in Mombasa, Lusaka, and Hawassa offer valuable insights into future QI training efforts in LMICs. By adapting content to fit local contexts, engaging multidisciplinary teams, and providing sustainable mentorship, QI courses may strengthen future healthcare QI efforts in resource‐limited settings.

## Author Contributions

**Anoosha Moturu:** conceptualization, methodology, project administration, investigation, data curation, writing – original draft, writing – review and editing. **Sehar Salim Virani:** methodology, investigation, data curation, formal analysis, writing – original draft, writing – review and editing. **Taylor Jaraczewski:** methodology, investigation, data curation, writing – review and editing. **Belay Melesse:** methodology, investigation, data curation, writing – review and editing. **Tien Vo:** methodology, investigation, data curation, writing – review and editing. **Clifford Y. Ko:** methodology, investigation, data curation, writing – review and editing. **Francis Pikiti:** methodology, investigation, data curation, writing – review and editing. **Hazel Sonkwe:** methodology, investigation, data curation, writing – review and editing. **Bright Moyo:** methodology, investigation, data curation, writing – review and editing. **Kelly McQueen:** data curation, methodology, investigation, writing – review and editing. **Girma Tefera:** data curation, methodology, investigation, writing – review and editing. **Xane Peters:** data curation, methodology, investigation, writing – review and editing. **Daniel Ojuka:** data curation, methodology, investigation, writing – review and editing. **Haytham Kaafarani:** data curation, methodology, investigation, writing – review and editing. **Robert Parker:** data curation, methodology, investigation, writing – review and editing. **Chris Dodgion:** data curation, methodology, investigation, writing – review and editing. **Deborah Rusy:** conceptualization, project administration, methodology, investigation, supervision, data curation, writing – review and editing. **Syed Nabeel Zafar:** conceptualization, project administration, methodology, investigation, supervision, writing – review and editing.

## Conflicts of Interest

The authors declare no conflicts of interest.

## Supporting information


**Table S1**: Lecture feedback from Hawassa and Lusaka course administrations.

## Data Availability

The data that support the findings of this study are available on request from the corresponding author. The data are not publicly available due to privacy or ethical restrictions.
