# Supplementary material for: Building Capacity in Perioperative Quality Improvement in Low‐ and Middle‐ Income Countries: Experiences From Mombasa, Lusaka and Hawassa
Source: World J Surg. 2025 Oct 9;50(1):75–84. doi: 10.1002/wjs.70126 (PMC12831525; doi:10.1002/wjs.70126)
Supplement: Supplementary file 1 — Table S1: Lecture feedback from Hawassa and Lusaka course administrations. [file WJS-50-75-s001.docx]

**Supplemental Table 1: Lecture Feedback from Hawassa and Lusaka Course Administrations**

*Usefulness – Likert scale – 1 Low, 5 High

*Volume of information – Likert scale – 1 Very Low, 3 Just the right amount, 5 Too much

*Speaker effective – Likert scale – 1 Low, 5 High

**Mean is reported for usefulness, volume of information and speaker effectiveness.*

*Table 1A: Hawassa lecture feedback*

| **HUSCH Session List** | **Session Usefulness** | **Volume of information** | **Speaker effectiveness** | **Would you recommend session to others?** |
| --- | --- | --- | --- | --- |
| Introduction to Healthcare Quality | 4.8 | 4.2 | 4.7 | Yes |
| Science of Improvement in the Healthcare Setting | 4.5 | 4.1 | 4.4 | Yes |
| Quality Improvement Models | 4.6 | 4.3 | 4.4 | Yes |
| Team dynamics & Problem solving  Team Formation and Functionality | 4.7 | 4.3 | 4.5 | Yes |
| KPI and clinical Audit | 4.8 | 4.4 | 4.7 | Yes |
| NSQIP and using data for surgical QI projects | 4.8 | 4.4 | 4.7 | Yes |
| Anesthesia data and QI from US perspective | 4.9 | 4.3 | 4.6 | Yes |
| Problem Identification, Prioritization | 4.8 | 4.1 | 4.6 | Yes |
| Problem Statement | 4.6 | 4.0 | 4.3 | Yes |
| Setting aim statement | 4.5 | 4.2 | 4.4 | Yes |
| Developing Change | 4.6 | 4.2 | 4.4 | Yes |
| Measurement | 4.9 | 4.3 | 4.4 | Yes |
| Test change idea and implementation | 4.8 | 4.1 | 4.6 | Yes |
| Sample QI project presentation | 4.8 | 4.2 | 4.4 | Yes |
| Small group discussions on new QI ideas | 4.7 | - | - | Yes |
| New QI projects presentations and action plan | 4.7 | - | - | Yes |
| **Overall feedback** | | | | |
| How do you value this workshop | | | | 4.7 |
| Would you recommend this workshop to colleagues? | | | | 3.0 |
| Did you like the venue? | | | | 4.4 |
| Was there enough breaktime? | | | | 3.8 |

*Table 1B: Lusaka lecture feedback*

| **UTH Detailed Session List** | **Session Usefulness** | **Volume of information** | **Speaker effectiveness** | **Would you recommend session to others?** |
| --- | --- | --- | --- | --- |
| Concept, Tools, and Methods for Quality Improvement | 4.6 | 3.6 | 4.5 | Yes |
| Quality improvement / Quality assurance in the Health Sector – strengthening systems | 4.6 | 3.7 | 4.5 | Yes |
| Importance of QI/QA in the delivery of surgical services in Zambia | 4.2 | 3.7 | 4.4 | Yes |
| Quality Care for maternal and newborn: lessons learnt from 7 facilities | 4.2 | 3.8 | 4.5 | Yes |
| Quality improvement initiatives in critical care nursing | 4.8 | 3.6 | 4.7 | Yes |
| Quality improvement for under-5 burns patients at UTH-Adult Hospital | 4.7 | 3.7 | 4.8 | Yes |
| Quality Improvement initiatives at UTH-Adult: Patient Waiting Times and Customer Satisfaction | 4.5 | 3.5 | 4.6 | Yes |
| Using data to monitor and improve processes | 4.6 | 3.8 | 4.7 | Yes |
| Perioperative registry and using data | 4.5 | 3.5 | 4.5 | Yes |
| Data and continuous improvement: Anaesthesia Perspectives | 4.6 | 3.7 | 4.6 | Yes |
| QI in perioperative nursing: Lessons Learnt | 4.4 | 3.8 | 4.3 | Yes |
| Embedding QI in residency programs | 4.7 | 3.5 | 4.7 | Yes |
| Examples of Resident QI projects / concepts | 4.7 | 3.6 | 4.6 | Yes |
| Breakout groups-Proposed QI projects Problem Identification, Prioritization and Aim Statement | 4.8 | 3.7 | 4.6 | Yes |
| **Overall feedback** | | | | |
| How do you value this workshop | | | | 4.9 |
| Would you recommend this workshop to colleagues? | | | | Yes |
| Would you have attended without any remuneration for transport or perdiem? | | | | Yes |
| Did you like the venue? | | | | 4.5 |
| Was there enough breaktime? | | | | 3.6 |
